# LETM presented with causalgia and ensued by sudden death

**DOI:** 10.1186/s12883-017-0791-8

**Published:** 2017-01-21

**Authors:** Rana Alnasser Alsukhni, Yasmin Aboras, Ziena Jriekh, Mahmoud Almalla, Ahmad Sheikh El-Kahwateya

**Affiliations:** 1 Division of Neurology, Department of Internal Medicine, Aleppo University Hospital, Aleppo, Syria; 2 Division of Rheumatology, Department of Internal Medicine, Aleppo University Hospital, Aleppo, Syria; 3 Department of Laboratory Medicine, Aleppo University Hospital, Aleppo, Syria

**Keywords:** LETM, RSD, NMOSD, Cervical spinal tumor

## Abstract

**Background:**

Longitudinally Extensive Transverse Myelitis LETM is a specific pattern of myelitis wherein at least three continuous vertebral segments are involved. Characteristically, it is a defining feature of neuromyelitis optica NMO. However, it is described in many other etiologies.

**Case presentation:**

We present a case of 60 year old male who presented with symptoms and signs of regional sympathetic dystrophy RSD followed by symptoms of myelitis. Spinal cord MRI revealed cervical LETM extending to the brainstem. In spite of serological negativity, treatment of suspected neuromyelitis optica spectrum disorder NMOSD was initiated and resulted in symptom relief. Meanwhile, sudden death occurred and autonomic dysreflexia was the main culprit.

**Conclusions:**

This case suggests that RSD could be the mere primary presentation of LETM, discusses the differential diagnoses of LETM in elderly patients, and suggests the possible risk of autonomic dysreflexia in such patients.

## Background

LETM is defined as an inflammation affecting the spinal cord and extending over three or more vertebral segments [1]. Seemingly, this definition is fully clear in terms of radiological characteristics, but far more ambiguous in terms of clinical presentation. Even though most of the related cases in the literature presented with symptoms of long tract dysfunction, more insidious presentations with causalgia were also conducted.

Reflex sympathetic dystrophy syndrome RSD is a clinical syndrome with three cardinal symptoms and signs; those are pain, edema and vasomotor dysfunction. The typical triggers are peripheral trauma, surgery and, to a lesser degree, central triggers like stroke and spinal cord injury, tumors and myelitis. In these cases, causalgia develops insidiously, months to years following the inducing pathology [2]. Interestingly, in 2011, Michael J. Regan and Jeetandera Rathi published a case report of cervical spinal cord tumor presented with causalgia [3]. Similarly, Asha Das conducted a case of a 53 year old female with bilateral optic neuritis presented with symptoms of left upper limb causalgia simultaneously with cervical spinal cord demyelination plaque which progressed later into syrinx [4].

A wide variety of pathologies may underlie LETM for it is described in the context of infectious, granulomatous, neoplastic and paraneoplastic diseases, acute disseminated encephalomyelitis (ADEM), spinal cord infarction, dural arteriovenous fistula, Sjogren syndrome and most commonly NMO and NMOSD.

NMOSD is a pretty new umbrella term used to include aquaporin-4 IgG antibodies AQP4-IgG seropositive patients with limited forms of NMO, those with typical NMO who have cerebral, diencephalon and brainstem lesions and AQP4-IgG seropositive patients with coexisting autoimmune diseases [5].

Since the definition of LETM depends mainly on radiological basis, many radiological characteristics were developed to imply certain pathologies, especially, NMO and NMOSD wherein the suggestive MRI characteristics are: involving spinal cord grey matter, association with cord swelling, central hypointensity on T1 weighted sequences, enhancement following IV gadolinium administration, and extension of a cervical lesion into the brainstem.

## Case presentation

A 60 year old nonsmoker male with no known personal or familial medical history presented to rheumatology clinic with a few days complaint of an odd feeling in the left hand with mild swelling and small joint arthralgia. The rheumatologist detected no explicit signs of arthritis. Nonetheless a week course of an oral NSAID was started. A week later, the patient came back with bilateral proximal upper limb weakness, finger past pointing and bilateral Babinski sign, and, although is not hypertensive, blood pressure measurements were 170/90, 160/90 respectively. He was accordingly referred to a neurology clinic.

The clinical history was reviewed and revealed no additional information. On inspection the patient was normal except for mild edematous left hand comparing with the right one. Blood pressure was normal in all of his visits to neurology clinic. Neurological examination showed intact HEENT except for bilateral weakness in trapezius and sternocleidomastoid muscles, bilateral asymmetric proximal arm and leg weakness and the left limbs were more affected than the right ones (Muscle strength was as follows upper left limb was 3/5 proximally and 4/5 distally, upper right limb was 3+/5 proximally and 4/5 distally, left lower limb was 4/5 proximally and 5/5 distally while lower right limb was 4+/5 proximally and 5/5 distally). Mild hypotonicity was noticed. Deep tendon reflexes DTRS were exaggerated all over and plantar response was extensor bilaterally. Apart from mild hypesthesia over the distribution of left C3 and C4 segments, superficial sensation was intact. However, proprioception was severely affected especially in the left arm with completely disappearance of stereognosis. Romberg sign was positive and tandem gait was intelligible. In the light of the pyramidal weakness, positive cerebellar signs were not suggestive. No symptoms of bladder or bowel dysfunction were detected.

## Localization

**Fig. 1:**
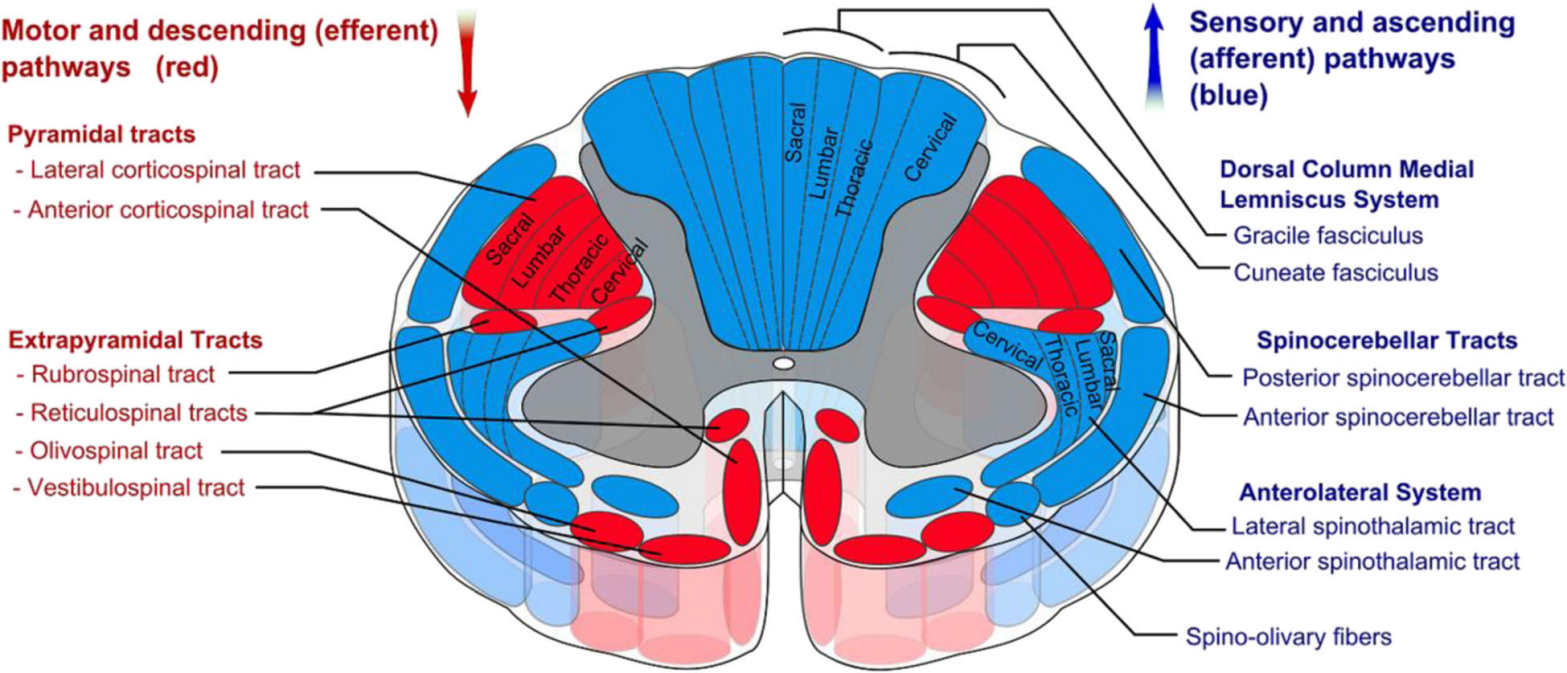
Transverse section of the spinal cord at the medcirvical level showing the general arrangement of the ascending tracts on the right and the descending tracts on the left. ‘https://commons.wikimedia.org/wiki/Category:Spinal_cord#/media/File:Spinal_cord_tracts_-_English.png’

Presence of tetraparesis with intact cranial nerves except for spinal root of 11th nerve along with brisk DTRS and Babinski sign localize the lesion to the upper cervical spinal cord. Brisk DTRs throughout the body place the lower limit of the lesion over cervical C5 spinal segment. Deeply affected proprioception with brisk DTRs indicates that posterior tracts of spinal cord are affected. Pyramidal signs indicate that corticospinal tracts are also involved, whereas the other tracts in the lateral columns of the white matter are relatively intact. Dysfunction of the spinal division of the accessory nerve may indicate that spinal grey matter is involved (Fig. 1).

**Fig. 2:**
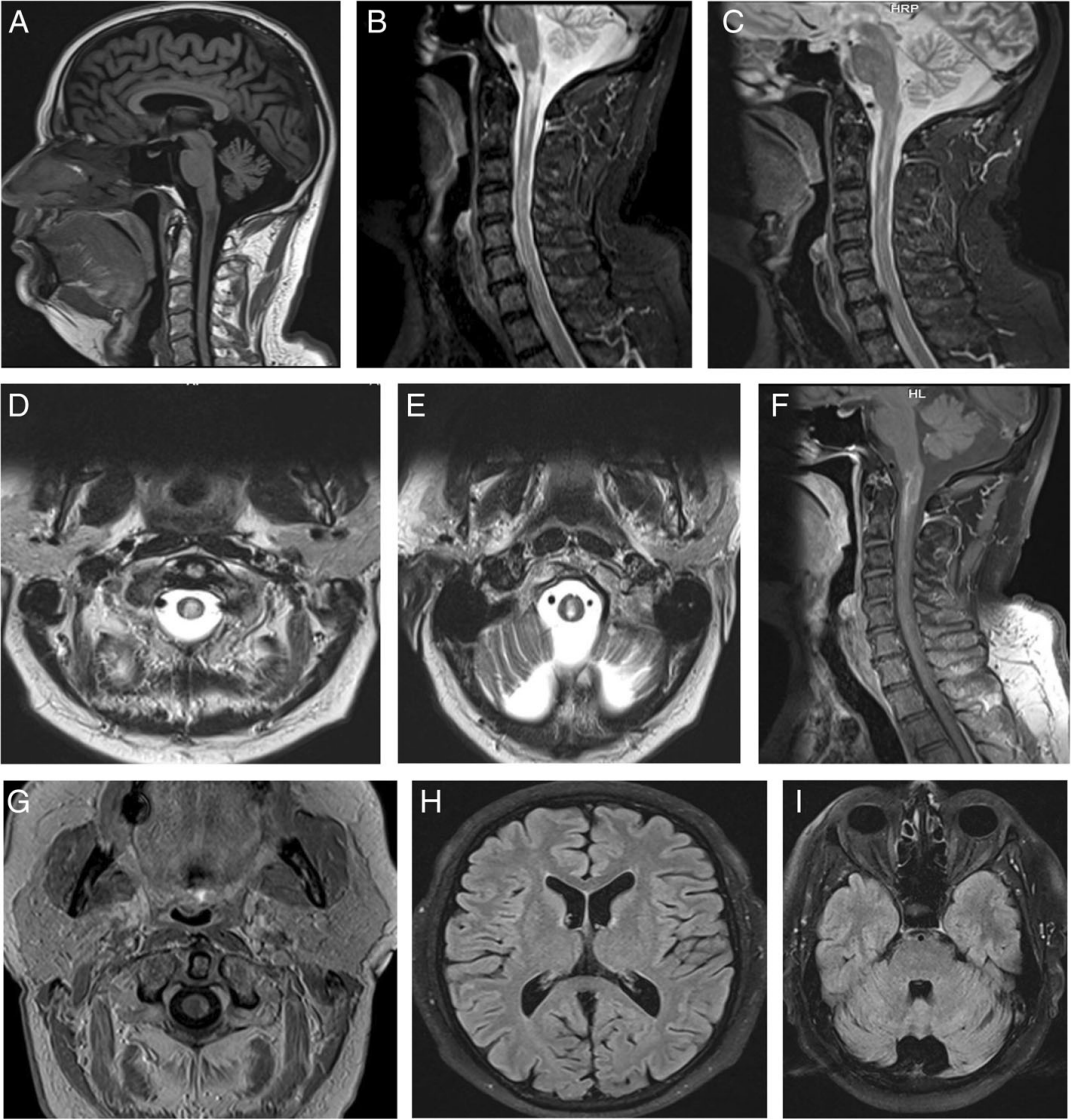
Spinal and cerebral MRI: **a** sagittal T1 image, **b** and **c** sagittal T2 images, **d** and **e** axial T2 images show spinal T1 hypointense and T2 hyperintense lesion extending over the first three cervical vertebrae and abutting medullary area postrema. **f** and **g** sagittal and axial T1 images with contrast show marginal enhancement. **h** and **i** axial flair images show normal cerebrum and cerebellum

Brain and cervical spinal cord magnetic resonance imaging MRI showed (Fig. 2).

Visually evoked potentials test VEP was unremarkable. Spinal tap revealed acellular fluid with normal glucose and a protein of 70 mg/dl. Cerebrospinal fluid CSF cytology showed acellular fluid.

Whole body computed tomography CT scan was normal.

Ordinary and autoimmune labs including antinuclear antibodies ANA, P and C anti neutrophil cytoplasmic autoantibodies ANCA, anti Sjogren’s syndrome-related antigen SS-A and B were all unremarkable.

**Fig. 3:**
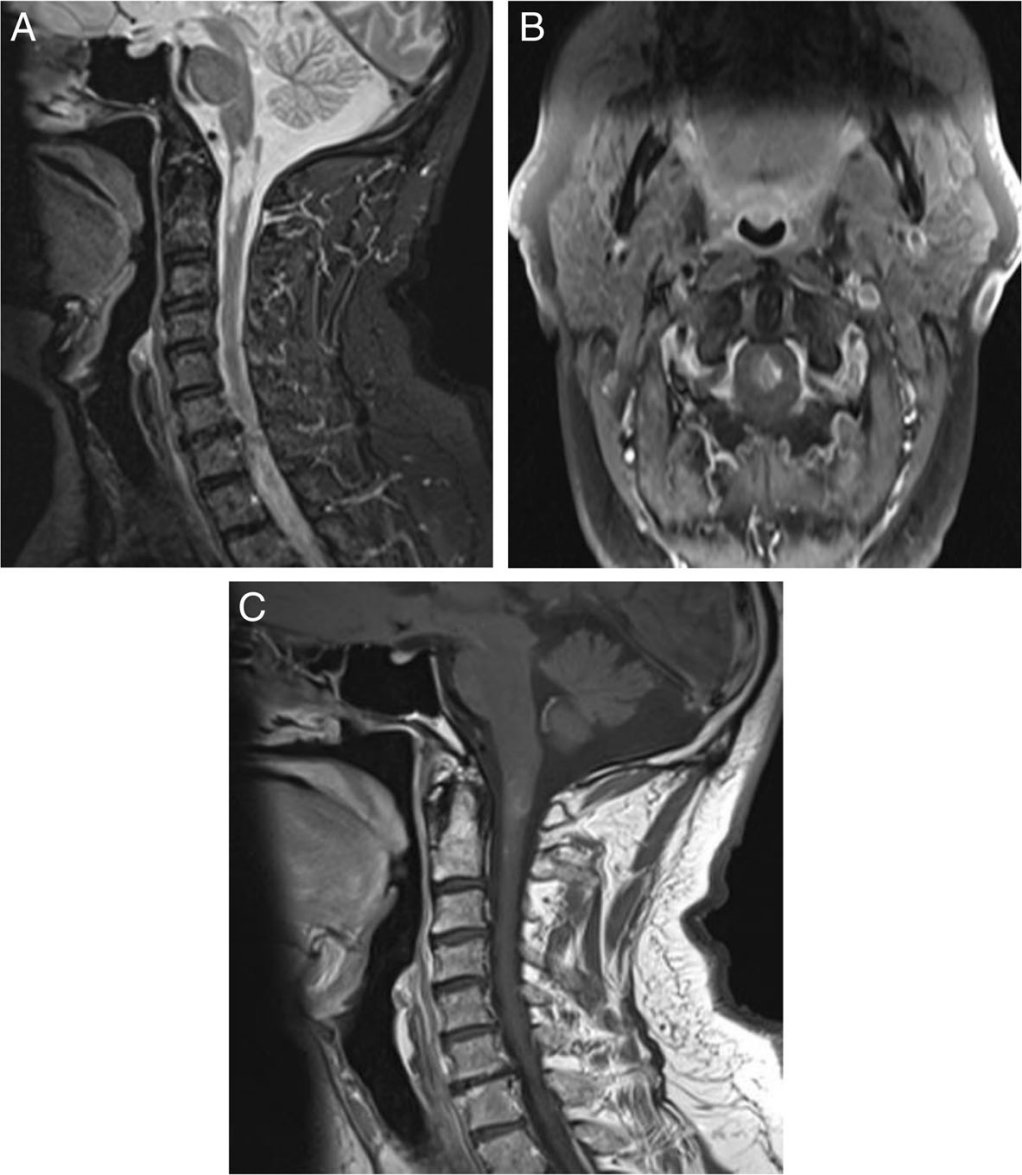
**a** sagittal spinal T2 image, **b** & **c** axial & sagittal spinal T1 images with contrast show the same previous changes with less demarcated contrast enhancement

Treatment with 1 g methyleprednisolone over 3 days followed by oral prednisolone 60 mg daily was started. Following the first dose of methyleprednisolone, the patient developed intractable hiccups which hardly responded to chlorpromazine. Two weeks later, the patient restored his normal motor abilities and Romberg sign became negative with normal tandem gait. However, limb spasticity progressed and left hand proprioception was not restored. During the following period, insidious dystrophic changes in left nails, hair and skin were developing with no pain. Moreover, two attacks of unexplained constipation and diarrhea complicated the course. A month from the presentation, cervical MRI with contrast was repeated and showed the same changes except from less demarcated enhancement comparing with the previous one (Fig. 3).

After that, AQP4 abs became available in other city, so that second spinal tap was done and revealed AQP4 antibodies negativity.

In spite of the recommendation with stereotactic biopsy, the patient refused and Azathioprine for suspected seronegative NMOSD was initiated. Each of complete blood count CBC, liver function tests LFTs were monitored weakly. Two weeks later, the patient suddenly felt dizzy, a seizure ensued and followed by sudden death.

## Discussion

Overwhelming pain is usually the mainstay feature of RSD. However, the pain in this patient was mild and limited to the acute phase of presentation and the other features accumulated during the following period. Although is not inherent in the presumed pathology of causalgia, superficial sensation is typically affected in most of the cases [6]. Hence, intact superficial sensation in the affected limb may represent another caveat in this case and may had a role in the absence of pain as a cardinal manifestation of RSD in this patient.

Prolonged list of differential diagnoses for LETM should have been excluded in this case starting with infectious diseases (Herpes viruses, TB), certain deficiencies including (B12 and copper Deficiency), vascular etiologies (spinal cord infarction and spinal cord arteriovenous shunt), neoplastic etiologies (lymphoma, astrocytoma and epindymoma) along with paraneoplastic etiologies in addition to autoimmune diseases including NMO, ADEM, multiple sclerosis MS, systemic lupus erythromatosus SLE, Sjogren syndrome, sarcoidosis and behcet disease [7].

Acellular CSF and absence of fever and other signs of inflammatory response made infectious causes unlikely. Moreover, the patient’s age, the absence of offending trigger, atypical radiologic features of the lesion and quick response to steroids weakened the possibility of nutritional deficiencies. The latter justification is also well applied for vascular causes which can only explain the acute presentation in elderly patient.

Paraneoplastic process does not typically present acutely or respond to steroids. Nonetheless, neither the above mentioned points nor unremarkable total body CT scan exclude this etiology. However, serum and CSF oligoclonal bands OCBs and anti neuronal paraneoplastic antibodies tests were not available.

Compared to each of neoplastic and paraneoplastic causes, Inflammatory disorders fit well and interpret both of the acute presentation and quick response to steroid. Negativity of serological autoimmune studies in this case added to inexistence of systemic signs and symptoms made LETM in the context of SLE, sjogren, sarcoidosis and behcet disease less possible.

Among other inflammatory disorders, preliminary NMO or NMOSD occupies core importance in this case since many radiological and some clinical signs advocate it. These include the acute presentation, intractable hiccups, responsiveness to steroid besides MRI features of cervical LETM extending to the medullary area postrema, affecting mainly spinal grey matter and enhancing with contrast. Although the age is not typical, this presentation meets most of the criteria of NMOSD without AQP4-IgG since the dissemination in space is missed and AQP4 abs test is negative [5].

CSF-IL6 was found to be elevated in NMO patients, [8] even in those with seronegative NMO, interestingly, this specific interleukin was also found to be elevated in RSD patients [9]. Unfortunately, titration of this interleukin was not applicable in this patient.

While the time required for radiological recovery is not well determined, fixed radiological features a month after presentation in spite of clinical amelioration and absence of cerebral demyelination lesions besides negative AQP4 abs may raise the possibility of underlying spinal tumor. Since negative cytology does not exclude spinal tumor, stereotactic biopsy would have settled the debate, but was unfortunately aborted by the patient’s refuse given the risk of this procedure in such location and the absence of an expert hand. Upon performing stereotactic biopsy in similar cases, demyelination rather than neoplastic process was revealed [10], a fact that led the authors to recommend an appreciation of the type of presentation (acute versus chronic) in determining the etiology, and a high index of suspicion of inflammatory process in acutely presenting cases [11].

Elevated CSF protein with normal CSF glucose and absence of cells may imply autoimmune etiology rather than neoplastic one, but can be simply explained by the block effect of the protruded disk at the level of C6, C7 vertebrae.

Sudden unexpected death was conducted in hospitalized patients with cervical spinal cord traumatic injuries and interpreted by autonomic dysreflexia [12]. Since this patient had no vascular risk factors, experienced no chest pain or dyspnea prior to his death and that neither prednisolone nor azathioprine is proved to cause sudden death, autonomic dysreflexia may be the culprit advocated by the highly located cervical lesion, his feeling of dizziness and the subsequent seizure just before the death. However, cardiac arrhythmia due to cardiac infarction or aortic dissection, or massive pulmonary embolism would be less possible.

Whether the unexplained high blood pressure on presentation, regional autonomic dystrophy and alternating constipation and diarrhea attacks hasten the possibility of underlying autonomic disturbance and a subsequent autonomic dysreflexia and sudden death is not known, but deserves to be considered in forthcoming cases.

Autonomic disturbances in patients with MS were the subject of many studies. The manifestations included sweating abnormalities, urinary dysfunction, cardiovascular and orthostatic dysregulation, gastrointestinal symptoms and sexual dysfunction. Cardiovascular autonomic disorders were in the form of fall in blood pressure, tachycardia, Postural orthostatic tachycardia syndrome (POTS), vasovagal syncope, while gastrointestinal dysregulation included constipation, fecal incontinence and dysphagia among other manifestations [13]. It is of Note that cardiovascular autonomic disorders were found to be more induced by brain stem lesions than spinal cord lesions in MS patients [14].

Autonomic disorders in NMO patients, however, are far less studies compared to those with MS. These disorders are considered either a part of hypothalamic syndrome [15], area postrema syndrome [16] or due to spinal lesions, and manifested as bladder and rectal problems [17], elevated blood pressure [18], fall in blood pressure, tachycardia and excessive sweating [19].

## Localization note

One would expect symptoms of syringomyelia in such patient, but the lesion extends to the white matter only at the levels of the first and second cervical vertebrae which correlates with spinal levels of C2 and C3, this may have affected sensory fibers of C3and C4 before or just after crossing to the opposite side and caused hypesthesia over these two dermatomes.

## Conclusions

In cases of incomplete presentation of arthritis, causalgia should be suspected and serial physical monitoring is recommended. In spite of its scarcity, autonomic dysreflexia may complicate highly located spinal lesions. Hints of autonomic disturbance may have an alarming role in this context and it may be beneficial to be checked early in the course.

## Abbreviations

ADEM: Acute disseminated encephalomyelitis
ANA: Antinuclear antibodies
ANCA: Anti neutrophil cytoplasmic autoantibodies
AQP4-IgG: Aquaporin-4 IgG
C: Cervical
CBC: Complete blood count
CSF: Cerebrospinal fluid
CT: Computed tomography
DTRS: Deep tendon reflexes
HEENT: Head, eyes, ears, nose and throat.
IV: Intravenous
LETM: Longitudinally Extensive Transverse Myelitis
LFTs: Liver function tests.
MRI: Magnetic resonance imaging
MS: Multiple sclerosis.
NMO: Neuromyelitis optica
NMOSD: Neuromyelitis optica spectrum disorder
RSD: Regional sympathetic dystrophy
SLE: Systemic lupus erythromatosus
SS-A and B: Anti Sjogren’s syndrome-related antigen
VEP: Visually evoked potentials test
